# Light Exposure at Night Disrupts Host/Cancer Circadian Regulatory Dynamics: Impact on the Warburg Effect, Lipid Signaling and Tumor Growth Prevention

**DOI:** 10.1371/journal.pone.0102776

**Published:** 2014-08-06

**Authors:** David E. Blask, Robert T. Dauchy, Erin M. Dauchy, Lulu Mao, Steven M. Hill, Michael W. Greene, Victoria P. Belancio, Leonard A. Sauer, Leslie Davidson

**Affiliations:** 1 Laboratory of Chrono-Neuroendocrine Oncology, Tulane University School of Medicine, New Orleans, Louisiana, United States of America; 2 Department of Structural and Cellular Biology, Tulane University School of Medicine, New Orleans, Louisiana, United States of America; 3 Tulane Cancer Center and Louisiana Cancer Research Consortium, New Orleans, Louisiana, United States of America; 4 Bassett Research Institute, Mary Imogene Bassett Hospital, Cooperstown, New York, United States of America; University of Texas Southwestern Medical Center, United States of America

## Abstract

The central circadian clock within the suprachiasmatic nucleus (SCN) plays an important role in temporally organizing and coordinating many of the processes governing cancer cell proliferation and tumor growth in synchrony with the daily light/dark cycle which may contribute to endogenous cancer prevention. Bioenergetic substrates and molecular intermediates required for building tumor biomass each day are derived from both aerobic glycolysis (Warburg effect) and lipid metabolism. Using tissue-isolated human breast cancer xenografts grown in nude rats, we determined that circulating systemic factors in the host and the Warburg effect, linoleic acid uptake/metabolism and growth signaling activities in the tumor are dynamically regulated, coordinated and integrated within circadian time structure over a 24-hour light/dark cycle by SCN-driven nocturnal pineal production of the anticancer hormone melatonin. Dim light at night (LAN)-induced melatonin suppression disrupts this circadian-regulated host/cancer balance among several important cancer preventative signaling mechanisms, leading to hyperglycemia and hyperinsulinemia in the host and runaway aerobic glycolysis, lipid signaling and proliferative activity in the tumor.

## Introduction

Renewed interest in the metabolism of cancer growth [1]–[3] has recently led to an intensive re-evaluation of the role played by aerobic glycolysis (e.g., the Warburg effect) in malignant growth progression [4]. In order to meet their bioenergetic needs to support the biosynthesis of the molecular and cellular building blocks required for rapidly increasing tumor biomass, cancer cells rely primarily on the Warburg effect rather than oxidative phosphorylation [1]–[4]. In fact, the Warburg effect is characterized by the robust cellular uptake of glucose and its metabolism to lactate via glycolysis in spite of an abundant supply of oxygen [1]–[4]. Studies have focused on signal transduction and transcriptional networks, including AKT [5], hypoxia-inducible factor-1 alpha (HIF-1α) and c-MYC [6] that drive the Warburg effect to redirect cancer cell bioenergetics towards the generation of molecular intermediates to support unrelenting cancer cell division [1]–[3]. Cancer cell proliferation and tumor growth also rely on the cellular uptake of linoleic acid (LA), an essential omega-6 fatty acid (FA) that is the most prevalent FA in the western diet [7]–[9]. In many tumors, cancer cells take-up LA via a cAMP-dependent transport mechanism and metabolize it to the mitogen 13-hydroxyoctadecadienoic acid (13-HODE) by the enzyme 15-lipoxygenase-1 [9]–[12], the activity of which is up-regulated by activation of epidermal growth factor (EGF) and insulin-like growth factor-1 (IGF-1) receptors [13], [14]. In many tumors, including human cancer xenografts [9], [11], [12], 13-HODE exerts a positive feedback effect on EGF and IGF-1 receptor growth signaling pathways to enhance downstream phosphorylation of ERK1/2 and AKT leading to amplified cell proliferation and survival responses [15]. It must be noted, however, that in some other cancer model systems and experimental contexts 13-HODE may play an antiproliferative role [16].

Homeostatic processes regulating mammalian physiology and metabolism in cells, tissues, and organs exhibit circadian rhythms that are synchronized by the light/dark cycle encompassing each 24-hour day. This is achieved through the endogenous oscillatory timekeeping activity of the SCN in the hypothalamus that receives information about the light/dark cycle from the eyes primarily via intrinsically photosensitive, melanopsin-positive retinal ganglion cells with additional input from rods and cones. Photic information is then processed by the SCN and transduced into an array of hormonal and neural outputs to peripheral target tissues [17]. In humans, disruption of the circadian regulation of metabolic processes including glucose and lipid metabolism induced via the abnormal timing of light may lead to an increased risk of type 2 diabetes, obesity [18], [19] and various malignancies including breast cancer as has been reported in night shift workers [20], [21]. The output signal of the SCN that most reliably reflects circadian activity is the nighttime pineal gland production of melatonin that is suppressed by LAN. The circadian melatonin signal has been linked to the temporal organization and synchronization of many physiological and metabolic activities including glucose and lipid metabolism [19], [22], [23].

Outputs from the SCN are believed to coordinate the biological timing of, and in the case of melatonin, modulate the processes governing tumor initiation, promotion and progression including cell proliferation, DNA synthesis, cell survival/apoptosis, cell cycle traverse, DNA damage/repair mechanisms, tumor suppressor activities and tumor cell invasion/metastasis [24]–[27]. We previously demonstrated that melatonin, at physiological nocturnal blood concentrations, directly inhibits tumor DNA synthesis and growth by suppressing the cAMP-dependent tumor uptake of LA and its metabolism to the mitogenic molecule 13-HODE [10], [12] via a melatonin receptor-mediated mechanism. However, the potential involvement of circadian regulation and the consequences of its disruption on cancer metabolism, particularly the Warburg effect, have never been addressed since investigations in this area have been based, almost exclusively, on *in vitro* studies in which the influence of cancer host central circadian regulation is absent. We postulated that the daily bioenergetic, metabolic signaling, and proliferative activities required for building tumor infrastructure exhibit dynamic circadian rhythms in both the host and tumor that contribute to tumor growth prevention. These rhythms, that are dependent upon the nighttime production of melatonin, a circadian tumor growth prevention signal, are disrupted by exposure of the host to LAN resulting in increased tumor metabolism and growth.

## Materials and Methods

### Ethics Statement

This study was carried out in strict accordance with the recommendations in the Guide for the Care and Use of Laboratory Animals of the National Institutes of Health. The protocol was approved by the Tulane University Institutional Animal Care and Use Committee (NIH Assurance # A4499-01). All animals were maintained in a facility accredited by the Association for the Assessment and Accreditation of Laboratory Animal Care, International. All surgery was performed under ketamine/xylazine anesthesia, and all efforts were made to minimize suffering.

### Reagents

High-performance liquid chromatography (HPLC)-grade chloroform, ethyl ether, methanol, glacial acetic acid, heptane, hexane, and Sep-Pak C18 cartridges for HPLC extraction of samples were purchased from Fisher Chemical (Pittsburgh, PA). Free fatty acid, cholesterol ester, triglyceride, phospholipid, rapeseed oil methyl ester standards, as well as boron trifluoride-methanol, potassium chloride, sodium chloride, perchloric and trichloroacetic acids were purchased from Sigma Scientific (St. Louis, MO). The HPLC standards, (+/−) 5-HETE and 13(S)-HODE), as well as UltraPure Water were purchased from Cayman Chemical Co. (Ann Arbor, Michigan).

### Animals, lighting conditions, tumor implantation, and growth

Weanling female nude rats (Hsd:RH-*Foxn1[rnu]*) between 35 and 50 g were housed in clear polycarbonate cages (2 rats/cage) in the laboratory facility light exposure chambers on a 12-hour light/12-hour dark (LD, 12∶12) cycle (lights on 0600–1800 hours or Zeitgeber time ZT0–ZT12 at 23°C and 45–50% humidity and allowed to acclimatize to these conditions for two weeks. Rats were provided water and food (Purina Prolab RMH 1000) ad libitum throughout the duration of the studies. Following the acclimatization period, animals were randomly separated into a control group of 36 rats that remained on the LD,12∶12 lighting regimen (complete darkness during the dark phase) and another group of 36 rats maintained on LD,12∶12 with exposure to dim LAN during the dark phase. The light intensity during the 12-hour light phase in both groups was provided by a one ballast/lamp system (GE Watt Miser, F34CW-RS-WM, 34-W bulb) in each chamber that provided a steady bright light stimulus at animal eye level of 141 µW/cm^2^ (345 lux). In the chamber providing dim LAN, there was a second ballast/lamp system (GE Starcoat, F32T8-SP-11, 32-W bulb) that emitted steady dim light stimulus at animal eye level of 0.08 µW/cm^2^ (0.2 lux) during the 12-hour dark phase [12]. Six weeks later, the animals were implanted with steroid receptor negative (SR−) MCF-7 human breast cancer xenografts in a tissue-isolated manner, as described previously [12]. These tumors had evolved over several passages from a subset of SR+ xenografts that had lost the expression of estrogen and progesterone receptors and became estrogen unresponsive. These xenografts were histopathologically characterized as poorly differentiated, grade 3, infiltrating ductal adenocarcinoma as previously described [12]. Over the course of circadian/tumor growth study, when tumors reached a sufficient size for measurement rats were subjected to light CO_2_ narcosis, and tumor dimensions were measured through the skin with vernier calipers every two days; tumor dimensions were converted to estimated tumor weights using a linear regression formula and growth rates calculated as previously described [9], [12]. The final tumor weight in the tumor growth study was determined by weighing at the end of the experiment. At the end of each of the tumor growth periods, when tumors reached approximately 5–6 g for both control and LAN-exposed groups, subsets of 6 animals were randomly killed and their corresponding tumors were harvested every 4 hours over a single 24-hour period at 6 different circadian time points (e.g., 0800 hours = ZT2, 1200 hours = ZT6, 1600 hours = ZT10, 2000 hours = ZT14, 2400 hours = ZT18, and 0400 hours = ZT22) as previously described [28]. Due to the greatly accelerated tumor growth rates in the LAN group, xenografts (n = 36) reached the targeted harvest size of 5–6 g approximately two weeks prior to that of the xenografts (n = 36) in the LD 12∶12 control group.

### Arterial Blood Collection from Tumor Host Rats

Following two weeks of the lighting regimens described above, animals were subjected to a series of six low-volume blood draws via cardiocentesis to collect left ventricular arterial blood, as described previously [9], [10], [12], [28] over a period of 30 days prior to tumor implantation (see above). Briefly, blood collections were performed at designated 4-hour intervals over a 24-hour period beginning at ZT2 (0800 hours) with each animal being subjected to cardiocentesis only once every 5 days during the 30-day period prior to tumor implantation. This was done in order for the animals to restore their small blood volume loss and to minimize stress and decrease the potential effects of the procedure on feeding and morbidity/mortality. Animals were lightly anesthetized by CO_2_ inhalation (70% CO_2_/30% air); 1-ml samples were taken from the left ventricle by cardiac puncture (less than 5% total blood volume) via tuberculin syringe (25 gauge, 3/8 in; Becton-Dickinson, Franklin Lakes, NJ) moistened with sodium heparin (1,000 U/ml; Elkin-Sinn, Cherry Hill, NJ), as described previously. Blood sampling during the dark-phase (i.e., 2000, 2400, 0400 hrs) was carried out under a safelight red lamp (Kodak 1A, model B, catalogue #152 1517; 120 V, 15 W, Rochester, NY) in order to preserve the nocturnal melatonin surge [9], [10], [12], [28]. Animal red lamp exposure at eye level during the brief 45-sec cardiocentesis procedure was no greater than 0.48±0.01 lux (1.16±0.04 µW/cm^2^). There were no complications due to anesthesia or cardiocentesis during the blood sampling period; survival was 100% and the animals were immediately active following the procedure. Plasma samples were stored at −20°C until assayed for melatonin, insulin, glucose, lactate and total fatty acids.

### Collection of Arterial Blood from Tumor Perfusion Donor Rats

Prior to initiation of the tumor perfusions, 3–4 adult nude female rats maintained on LD 12∶12 were anesthetized using ketamine-xylazine solution (89.1 mg/kg and 9.9 mg/kg, IP). Animals were heparinized via jugular injection of sodium heparin (25 mg/kg; Sargent Pharmaceuticals, Schaumburg, IL). Forty to 50 mls arterial blood was collected between 0600 and 0900 hrs (low melatonin levels) from the right carotid artery of donor rats via catheter for the tumor perfusion experiments (3 to 4 perfusions per group), pooled, filtered through a 2″×2″ cheesecloth pad (Kendall Curity Gauze Sponge; Tyco Healthcare, Mansfield, MA), stored in a reservoir under mineral oil (Cat. #M1180-500 ML; Sigma Scientific, St. Louis, MO) and chilled at 4°C in a reservoir on ice and gently mixed via mechanical stirrer (Model #6975-171; Corning, NY) [9], [10], [12], [28].

### Tissue-Isolated Tumor Perfusion *In Situ*

When (SR−) MCF-7 human breast cancer xenografts reached 5–6 gm estimated tumor weight, animals were prepared for arterial-venous difference measurements of total fatty acids (TFAs), LA, 13-HODE, glucose, lactate, pO_2_, CO_2_ and pH between 0600 and 1000 hrs when endogenous melatonin levels were low. Tissue-isolated xenografts were perfused *in situ* with rat donor whole-blood to which either synthetic melatonin (Sigma, St. Louis, MO) and/or 13(S)-HODE (Cayman Chemicals, Ann Arbor, MI), or MT_1_/MT_2_ melatonin receptor antagonist S20928 (a generous gift from Institute de Recherches Internationales Servier, Courbevoie Cedex, France) was added as previously described [9], [10], [12], [28]. Sets (3 or 4 tumors/perfusion) of tissue-isolated human breast cancer xenografts were perfused *in situ* for 1 hr as previously described [9], [10], [12], [28] with whole-blood collected from donor rats. Incorporation of [^3^H]thymidine into tumor DNA was initiated 20 minutes prior to the end of each perfusion by injecting 20 µl of physiological saline containing 2 µCi [methyl-^3^H]thymidine/gm (New England Nuclear, Perkin Elmer, Boston, MA) estimated tumor weight into the arterial catheter. At the completion of each perfusion, tumors were freeze-clamped under liquid nitrogen, weighed and store at −85°C until analysis.

### Arterial Glucose, Lactate and Acid/Gas Measurements

During the course of this study arterial whole blood samples were taken for measurements of pH, pO_2_, pCO_2_, glucose and lactate levels, and hematocrit using an iSTAT1 Analyzer and CG4+ and CG8+ cartridges (Abbott Laboratories, East Windsor, NJ). Values for glucose and lactate are reported as mg/dL and mmol/L, and for pO_2_ and pCO_2_ as mm Hg. Minimum detection levels for pH, pO_2_ and pCO_2_, glucose and lactate values were, respectively, 0.01, 0.1 mm Hg, 0.1 mm Hg, 0.2 mg/dL and 0.01 mmol/L.

### Melatonin and insulin analyses

Arterial plasma melatonin levels were measured via radioimmunoassay using the melatonin rat ^125^I – radioimmunoassay kit (Labor Diagnostika Nord, Nordham, Germany) and analyzed using a Packard Cobra 5005 Automated Gamma Counter (Palo Alto, CA), as previously described [10], [12]. Arterial plasma insulin levels were measured using an ELISA kit (Diagnostic Systems Labs., Webster, TX).

### Fatty acid extraction and analysis

Arterial plasma free fatty acids (FFA), triglycerides (TGA), phospholipids (PL), and cholesterol esters (CE) were extracted from 0.1 ml samples, as previously described [9]–[10], [12], [28]. Prior to extraction heptadecanoic acid (100 µg), which had been dissolved in chloroform (Fisher Scientific, Fair Lawn, NJ), was used as an internal standard. Methyl esters of fatty acids (FAs) were analyzed using a Hewlett Packard (Palo Alto, CA) model 5890A Gas Chromatograph fitted with a flame ionization detector (model #7673 A) auto-injector (model #7673 S), and integrator (model #3396A). All separations were carried out using a 0.25-mm×30-m capillary column (model #2380; Supelco, Inc., Bellefonte, PA) at 190°C, with helium as the carrier gas (linear rate 20 cm/s; split 100∶1). Injection port and detector were adjusted to 220°C. All methyl esters were identified on the basis of their retention time, compared with that of known standards. Minimum detectable limit for the assay was 0.05 µg/ml.

### Tumor lysate extraction, protein extraction and Western blot analysis

Frozen tumors were pulverized and manually homogenized in 50 mM Hepes, pH 7.5, 150 mM NaCl, 1% NP-40, 0.1% SDS, 0.1% sodium deoxycholate, 1 mM Na3VO4, 100 nM okadaic acid, and 1× protease inhibitor cocktail Set I (Calbiochem/EMD Biosciences, Billerica, MA). Tumor lysates were centrifuged at 15,300×g for 20 minutes. Supernatants were aliquoted and stored at −80°C. Protein concentrations of the supernatants were determined using a protein assay kit (Bio-Rad Laboratories, Inc., Hercules, CA). Total protein (80 µg per sample) was electrophoretically separated on a 10% SDS-polyacrylamide gel and electroblotted onto a Hybond membrane. After incubation with 5% non-fat milk in Tris-buffered saline containing 0.1% Tween, the immunoblots were probed with antibodies to phospho-AKT (Ser^473^), c-MYC (Cell Signaling Tech., Inc., Danvers, MA) or HIF-1α (BD Biosciences, San Jose, CA). The same blots were stripped and re-probed with antibodies to AKT (Cell Signaling Technology, Inc.), tubulin or GAPDH (glyceraldehyde-3-phosphate dehydrogenase) (Millipore Corp., Billerica, MA).

### Statistical Analysis

The presence of significant tumor rhythms over the 24-hour period and the degree of their robustness in either the circadian-regulated control or –disrupted LAN-exposed groups were determined by cosinor analysis using Periodogram (VBScript) software. Raw individual tumor data collected over a single 24-hour period were analyzed by this method with a fixed 24-hour period. Statistical differences between mean values in the LAN-exposed group versus the control group at each circadian time point were assessed using an unpaired Student’s t test. Statistical differences among group means in the tumor perfusion studies were determined by a one-way ANOVA followed by Bonferroni’s Multiple Comparison Test to make the following multiple comparisons among all groups: control versus melatonin; control versus melatonin +13-HODE; control versus 13-HODE; melatonin versus melatonin +13-HODE; melatonin +13-HODE versus 13-HODE; and melatonin versus 13-HODE. Differences in the slopes of regression lines (i.e., tumor growth rates) among groups were determined by regression analyses and tests for parallelism (Student’s t test). Differences were considered to be statistically significant at p < 0.05. Student’s t test, one-way ANOVA followed by Bonferroni’s post hoc test, and linear regression analyses were all carried out using GraphPad Prism 5 software.

## Results

### Circadian regulation and disruption of host circulating factors

**Figure 1:**
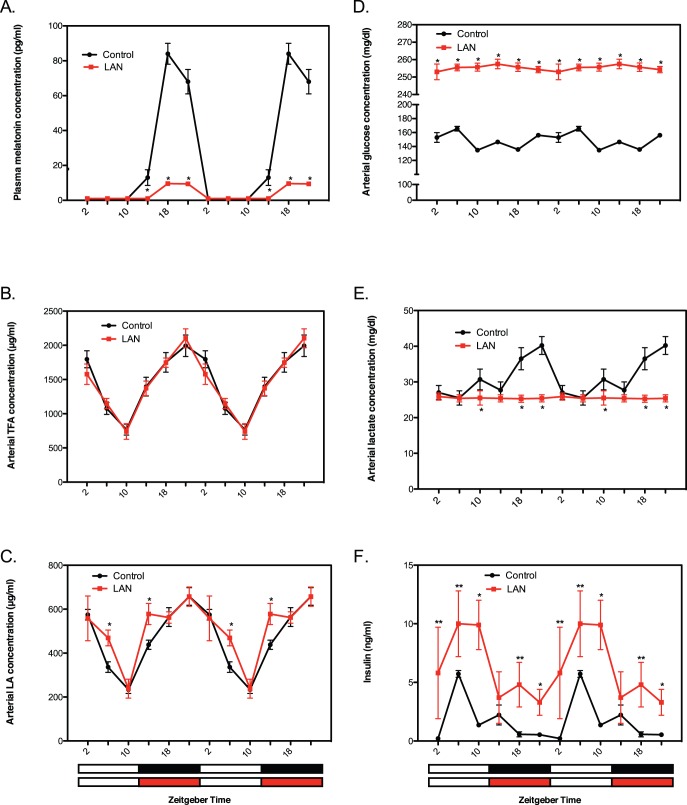
Circadian oscillations and their LAN-induced circadian disruption in cancer host levels of hormonal, metabolic and dietary factors. (**A–F**) Plasma levels of melatonin (A), TFAs (B), LA (C), glucose (D), lactate (E) and insulin (F) were measured in nude female host rats bearing tissue-isolated SR- MCF-7 human breast cancer xenografts under LD,12∶12 (solid black circles) or LD,12∶12+ LAN (0.2 lux) (solid red circles). Arterial blood samples were obtained via cardiac puncture at six different circadian time points during a single 24-hour period. The same single 24-hour pattern for each plasma analyte is displayed twice to illustrate rhythm continuity. Zeitgeber time (ZT) represents hours after lights on (ZT0 or 0600 hours); lights off at ZT12 (1800 hours). Black bars at bottom of figures indicate the dark phase and red bars indicate LAN. Solid black or red circles are mean ±SD; n = 6 at each time point. Significant rhythmic patterns under LD,12∶12 conditions in A – F, p<0.001; significant but disrupted rhythmic patterns under dim LAN conditions A – C and F only, p<0.001 (one-way ANOVA). *p<0.01; **p<0.05 LAN vs LD,12∶12 (Student’s *t* test).

We first examined potential circadian dynamics and their disruption by LAN in a number of host factors including plasma levels of melatonin, total FAs (TFAs), LA, glucose, lactate and insulin in rats bearing tissue-isolated, steroid receptor negative (SR−) MCF-7 human breast cancer xenografts on an light/dark (LD) 12∶12 cycle [12]. Plasma melatonin levels were low throughout the light phase and markedly increased to peak amplitude in the middle of the dark phase (Fig. 1A). Exposure of animals to dim LAN induced an 88% suppression in the nocturnal melatonin amplitude without affecting its circadian phase (Fig. 1A). Plasma TFA (Fig. 1B) and LA (Fig. 1C) rhythms in LD controls, which are dependent upon circadian SCN-driven nocturnal feeding activity, were preserved in LAN-exposed rats indicating that circadian disruption was limited to suppression of the nocturnal melatonin signal. Circadian control of both blood glucose and insulin levels independent of the effects of food intake has been reported in rats [29]. Plasma glucose levels increased after lights on to peak at the mid-light phase followed by a decline until lights off (Fig. 1D). Following lights off, glucose concentrations initially increased then decreased to a nadir at the mid-dark phase followed by a second increase during the end of the dark period. Plasma lactate concentrations in animals maintained in LD 12∶12 were generally low throughout the light phase followed by an increase during the dark period to reach a peak two hours before lights on (Fig. 1E). Under dim LAN conditions, rats became hyperglycemic with arrhythmic blood glucose levels remaining markedly elevated at 50% above control levels (Fig. 1D) whereas arrhythmic blood lactate levels remained consistently lower than controls throughout the 24-hour period (Fig. 1E). Circulating insulin levels exhibited a daily oscillation under LD 12∶12 (Fig. 1F) that generally mirrored the pattern of circulating IGF-1 concentrations previously reported [28]. LAN-exposed rats became markedly hyperinsulinemic with insulin levels retaining an oscillatory pattern similar to that observed in the LD 12∶12 controls (Fig. 1F); circulating IGF-1 levels were also circadian-disrupted and elevated under LAN conditions [28].

### Circadian regulation and disruption of tumor glucose and linoleic acid metabolism

**Figure 2:**
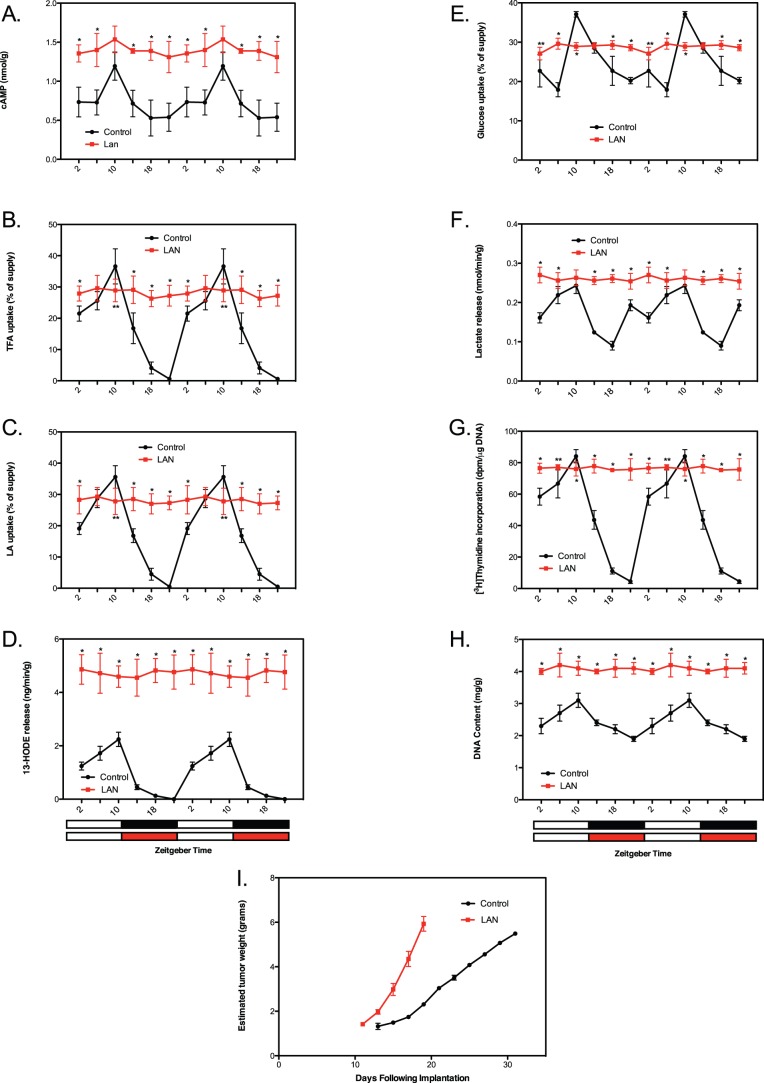
Circadian oscillations and their LAN-induced disruption in tumor cAMP signaling, fatty acid uptake and metabolism, the Warburg effect and proliferative activity and their impact on tumor growth. (**A–H**) Tumor levels of cAMP (A), TFA uptake (B), LA uptake (C), 13-HODE formation (D), glucose uptake (E), lactate formation (F), [^3^H]thymidine incorporation into DNA (G), and DNA content were measured under LD,12∶12 or LD,12∶12+ LAN; see legend for Figure 1 for further experimental conditions. (**I**) Tumor growth in both groups was measured over the course of the experiment. Solid black or red circles are mean ±SD estimated tumor weight; n = 6 for estimated tumor weight. Cosinor analysis revealed robust and highly significant rhythmic patterns under LD,12∶12 conditions in tumor analytes; no significant daily rhythmic patterns were detected under dim LAN (see summary Table 3). *p<0.01; **p<0.05 LEN vs LD,12∶12 (Student’s *t* test). Tumor growth curves in I, p<0.01 slope of tumor growth in LAN group vs LD, 12∶12 group (linear regression).

We next tested whether tumor xenografts themselves exhibited circadian rhythms in tumor cAMP levels, LA uptake, 13-HODE formation and the Warburg effect that could be disrupted by LAN-induced melatonin suppression. In the present study, the Warburg effect is identified as the tumor uptake of arterial blood glucose coupled with the release of lactate into the tumor venous blood [1]–[4]. Under LD, 12∶12 conditions, tumor cAMP levels (Fig. 2A), TFA uptake (Fig. 2B), LA (Fig. 2C) uptake, and 13-HODE formation (Fig. 2D), increased during the light phase to reach a peak two hours before lights off followed by a decrease during the dark phase to reach a nadir two hours before lights on. Tumor glucose uptake (Fig. 2E) and lactate production (Fig. 2F) followed a similar daily rhythmic pattern by increasing during the light phase to reach a peak two hours prior to lights off. During the dark period there was a progressive decline in these parameters with glucose uptake reaching a nadir at the end of the dark phase and lactate production decreasing to a trough at the mid-dark period followed by a rise until two hours before lights off. Under LAN conditions, however, not only were these rhythms completely absent, but the levels of each of these parameters remained significantly elevated over the 24-hour period.

### Circadian regulation and disruption of tumor expression of signaling and transcriptional factors

**Figure 3:**
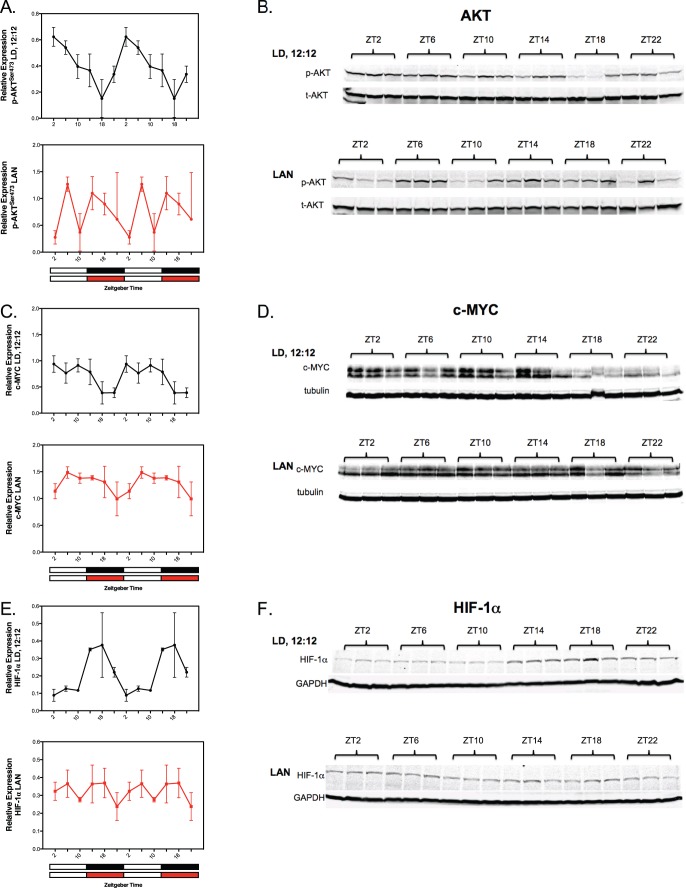
Circadian oscillations and their LAN-induced circadian disruption in tumor signaling and transcriptional regulatory molecules involved in the Warburg effect (e.g., AKT, c-MYC and HIF-1α). (**A–F**) Tumor levels of phospho-AKT^ser473^ (A & B), cMyc (C & D), and HIF-1α (E & F) were measured under LD,12∶12 or LD,12∶12+ LAN; see legend for Figure 1 for further experimental conditions. Solid black or red circles represent the mean ±SD relative expression (derived from the densitometric quantitation of the immunoblots) of either pAKT^ser473^, c-MYC and HIF-1α at each circadian time point; n = 3 representative tumor samples at each time point. Relative expression of pAKT^ser473^ represents the ratio of pAKT^ser473^ protein to total (t)AKT protein; relative expression of c-MYC and HIF-1α represents the ratio of these proteins to tubulin and GAPDH, respectively. Cosinor analysis revealed robust and highly significant rhythmic patterns under LD,12∶12 conditions in tumor analytes; except for c-MYC, no significant daily rhythmic patterns were detected under dim LAN (see summary Table 4). Statistical comparisons were unable to be made between corresponding time points between the LD,12∶12 and LAN groups since immunoblots were performed for the two groups in separate runs. Only a single 24-hour cycle of immunoblots were run and displayed, whereas the densitometric representation of those same immunoblots was displayed twice to illustrate rhythm continuity as indicated in the legend of Fig. 1.

We also addressed whether AKT, c-MYC and/or HIF-1α, key transcriptional regulators of the Warburg effect, exhibit circadian rhythmicity that is disrupted by dim LAN. As a pivotal stimulatory signal for tumor cell survival/proliferation, and glucose and LA metabolism, AKT activation [e.g., phosphorylation at serine 473 (s473)] was highest during the light phase and decreased thereafter to a nadir during the mid-dark phase. HIF-1α expression levels were low during the light phase followed by an increase to peak expression during the dark phase (Fig. 3 E&F). However, under dim LAN exposure, the rhythmic expression of both phospho-AKT (pAKT^s473^) and HIF-1α was completely disrupted (Fig. 3 A&B and E&F). Tumor c-MYC expression was greatly elevated during the entire light phase followed by a marked decrease to a nadir during the second half of the dark phase (Fig. 3 C&D). In response to LAN-induced melatonin suppression, however, a significant, but less robust rhythmic expression of c-MYC persisted over the 24-hour period (Figs. 3 C E&F).

### Circadian regulation and disruption of tumor proliferative activity

In light of the above findings, we postulated the occurrence of corresponding circadian changes in tumor proliferative activity and DNA content. We found that tumor [^3^H]thymidine incorporation into DNA (Fig. 2G) as well as DNA content (Fig. 2H) increased during the light phase shortly after lights on to reach a peak two hours prior to lights off. During the dark phase, however, both [^3^H]thymidine incorporation and DNA content decreased to a nadir two hours before lights on (Figs. 2 G&H). The circadian oscillations in [^3^H]thymidine incorporation and DNA content suggest that tumor cell number increased during the light phase due to increased proliferative activity while during the dark phase cell number decreased suggesting that apoptotic cell loss was occurring in parallel with decreased cell proliferation. Our results are consistent with previous studies in transplantable mouse mammary cancer showing that gross tumor sizes and growth rates actually fluctuate in a circadian manner throughout each day of the period of net tumor growth [30]. In response to LAN, the daily oscillation in proliferative activity and DNA content was nullified as these parameters remained persistently elevated throughout the 24-hour period (Fig. 2 G&H). Under circadian-regulated conditions, tumor growth increased steadily over a two-week period whereas under circadian-disrupted conditions the growth rate increased by two-fold over the controls (Fig. 2I).

### Potential melatonin regulation of the Warburg effect via inhibition of 13-HODE activation of AKT

**Figure 4:**
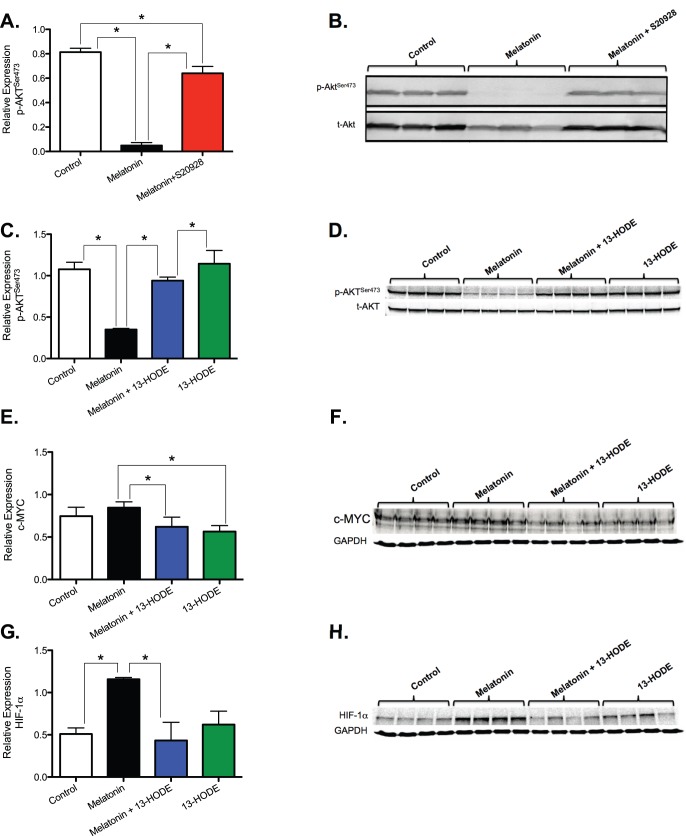
Response of signaling and transcriptional regulatory factors involved in the Warburg effect (e.g., AKT, c-MYC and HIF-1α) in tissue-isolated SR- MCF-7 human breast cancer xenografts to short-term perfusion with nocturnal physiological concentrations of melatonin either in the absence or presence of melatonin receptor antagonist S20928 or 13-HODE. Xenografts were perfused *in situ* for 60 min with rat donor whole blood containing either vehicle, melatonin (500 pM or 1 nM), S20928 (1 nM), 13-HODE (12 µg/ml), melatonin+S20928 or melatonin +13-HODE. (**A – H**) Tumor perfusions were performed between 2–4 hours after lights on (0600 hours) under LD,12∶12 conditions. In the perfusions A & B, a melatonin concentration of 1 nM was used whereas in all other perfusions (C – H) melatonin was used at 500 pM. Vertical bars represent the mean (±SD) relative expression (derived from the densitometric quantitation of the immunoblots) in tumors (n = 3–4) of either pAKT^s473^ (A–D), c-MYC (E & F), or HIF-1α (G & H). Relative expression of pAKT^ser473^ represents the ratio of pAKT^ser473^ protein to total (t)AKT protein; relative expression of c-MYC and HIF-1α represents the ratio of these proteins to GAPDH. Statistical analysis was performed using one-way ANOVA followed by Bonferroni’s Multiple Comparison Test to make multiple comparisons among all groups indicated by the brackets within each bar graph: * over each bracket indicates that differences are statistically significant at p<0.05.

We next took the first preliminary step to elucidate a plausible mechanism by which the host circadian system regulates circadian tumor LA uptake/metabolism, aerobic glycolysis, signaling and growth via melatonin-induced inhibition of 13-HODE formation and the ability of 13-HODE to activate AKT. As a first step, we tested whether a high physiological nocturnal concentration of melatonin would decrease the expression of phospho-Akt (pAKT^s473^) via a melatonin receptor-mediated mechanism. A one hour perfusion of tissue-isolated human breast cancer xenografts *in situ* with rat whole blood containing melatonin (1 nM) resulted in a down-regulation of pAKT^s473^ accompanied by a marked reduction in total AKT protein. These effects were prevented by tumor co-perfusion with non-selective MT_1_/MT_2_ melatonin receptor antagonist S20928 indicating that melatonin-induced down-regulation of pAKT^s473^ was melatonin receptor mediated (Fig. 4 A&B) most likely via the MT_1_ melatonin receptor [12], [27]. These results are consistent with the postulate that melatonin inhibits LA uptake, 13-HODE formation and proliferative activity in tissue-isolated human breast cancer xenografts via suppression of AKT phosphorylation activity.

Since a physiological nocturnal melatonin concentration, reduces both p-Akt^s473^ and 13-HODE formation, we proposed that the elevated production of 13-HODE during the light phase may serve as an important new fatty acid metabolic signaling link to activation of the Warburg effect via its ability to activate AKT in cancer cells. During the dark phase, melatonin may indirectly inhibit the Warburg effect via its suppression of 13-HODE formation and subsequently its ability to stimulate aerobic glycolysis via activating AKT. To test this postulate, xenografts were perfused for one hour *in situ* with a lower physiological nocturnal concentration of melatonin (500 pM) in the absence or presence of 13-HODE followed by the assessment of pAKT^s473^, c-MYC and HIF-1 αexpression. Melatonin down-regulated pAkt^s473^ (Fig. 4 C&D), had no effect on c-MYC (Fig. 4 E&F) and up-regulated HIF1-α expression (Fig. 4 G&H). The lack of an acute effect on c-MYC may have been due to the inability of a short perfusion time to influence c-*myc* gene expression. Melatonin also significantly suppressed tumor cAMP levels, LA uptake, 13-HODE production, the Warburg effect, (e.g., reductions in tumor glucose uptake, lactate release, O_2_ uptake and CO_2_ production), [^3^H]thymidine incorporation and DNA content; co-perfusion of melatonin with 13-HODE negated these effects (Tables 1 and 2).

**Table 1:** Tumor glucose uptake and lactate release (Warburg effect), arterial and venous oxygen (O_2_) and carbon dioxide (CO_2_), tension O_2_ uptake and CO_2_ production measured across MCF-7(SR−) human breast cancer xenografts perfused for 60 minutes with rodent donor whole blood *in situ.*

Treatment	Glucose uptake(nmol/min/g)	Lactate release(nmol/min/g)	pO_2_ Artery/Vein(mm Hg)	O_2_ uptake(% of supply)	pCO_2_ Artery/Vein(mm Hg)	CO_2_ production(% of original value)	pH Artery/Vein
**Controls**	20.5±1.4 (25.6±1.4%)†	29.1±1.4	139.7±.9/55.6±2.8**	61.0±2.0	29.0±1.9/63.3±1.5**	119.0±4.0	7.44±0.01/7.31±0.01**
**Melatonin (500 pM)**	11.1±1.7* (14.3±2.0%)* †	18.0±1.2*	145.2±1.0/67.6±3.5**	52.0±3.0*	31.0±0.4/58.5±2.1**	89.0±7.0*	7.43±0.01/7.31±0.01**
**Melatonin +13-HODE (12 µg/ml)**	22.1±2.1 (29.7±0.1%)†	24.4±0.4	142.0±5.0/54.8±3.4**	61.0±2.0	30.5±1.9/65.9±2.7**	117.0±6.0	7.42±0.02/7.32±0.01**
**13-HODE**	19.9±3.7 (28.1±3.2%)†	28.0±2.1	142.2±5.4/52.9±2.6**	63.0±2.0	30.6±1.0/67.8±1.9**	119.0±11.0	7.42±0.01/7.31±0.01**

Donor blood contained either melatonin, 13-HODE, or melatonin plus 13-HODE. Values are means ± SD (n = 4/group).

*p<0.01 vs. Control and Melatonin +13-HODE.

**p<0.01 vs. arterial value.

† Values expressed as % supply.

**Table 2:** Tumor cAMP levels, TFA uptake, LA uptake, 13-HODE formation, [^3^H]thymidine incorporation into DNA and DNA content in tissue-isolated MCF-7 (S−) human breast cancer xenografts perfused for 60 minutes with rodent whole blood *in situ*.

Treatment	cAMP(nmol/g tissue)	TFA uptake(mg/min/g)	LA uptake(mg/min/g)	13-HODE Release(ng/min/g)		[^3^H]thymidine incorporation(dpm/mg DNA)	DNA content(mg/g)
				Arterial Supply	Venous Output		
**Controls**	0.58±0.08	3.46±0.83 (22.1±2.6%)†	0.95±0.13 (21.2±3.2%)†	0	1.82±0.34	53.4±3.1	2.5±0.2
**Melatonin (500 pM)**	0.22±0.07*	0	0	0	0	6.3±1.0*	1.9±0.1*
**Melatonin +13-HODE** **(12 µg/ml)**	0.90±0.08**	0	0	415.8±26.9	321.8±19.4**	87.0±3.2**	2.7±0.1**
**13-HODE**	0.92±0.09**	3.02±0.49 (21.8±1.4%)†	0.99±0.06 (22.6±1.9%)†	430.9±22.7	330.2±16.3**	89.2±2.5**	2.8±0.1**

Donor blood contained either melatonin, 13-HODE or melatonin plus 13-HODE. Values are mean ± SD (n = 4/group).

*p<0.01 vs. Controls and Melatonin +13-HODE.

**p<0.01 vs. Controls.

† Values expressed as % supply.

**Table 3:** Circadian oscillations and their LAN-induced disruption in tumor cAMP signaling, fatty acid uptake and metabolism, the Warburg effect and proliferative activity and their impact on tumor growth.

	LD,12∶12		LAN	
Analyte	Robustness*	P-Value†	Robustness*	P-Value†
**cAMP levels**	37.1%	0.000251	0.0%	NS
**Linoleic Acid uptake** **	92.4%	<0.000001	0.0%	NS
**13-HODE production**	86.1%	<0.000001	0.0%	NS
**Glucose uptake**	38.2%	0.000206	5.4%	NS
**Lactate production**	51.3%	0.000025	0.0%	NS
**Thymidine incorporation**	89.9%	<0.000001	0.0%	NS
**DNA content**	75.4%	<0.000001	0.0%	NS

*Robustness is the percent of variance explained by the fitted cosine model.

**Total fatty acid uptake cosinor analysis recapitulated linoleic acid uptake results (data not shown).

† P-Values are for cosinor analysis performed on raw individual data with a fixed 24-hr period; n = 36 for LD,12∶12 and n = 36 for LAN for each analyte.

**Table 4:** Circadian oscillations and their LAN-induced circadian disruption in tumor signaling and transcriptional regulatory molecules involved in the Warburg effect (e.g., AKT, c-MYC and HIF-1α).

	LD,12∶12		LAN	
Analyte	Robustness*	P-Value†	Robustness*	P-Value†
**AKT expression**	35.5%	0.000920	0.0%	NS
**cMYC expression**	36.5%	0.008501	25.6%	0.027081
**HIF1α expression**	60.5%	0.000419	0.0%	NS

*Robustness is the percent of variance explained by the fitted cosine model.

† P-Values are for cosinor analysis performed on raw individual data with a fixed 24-hr period; n = 36 for LD,12∶12 and n = 36 for LAN for each analyte.

## Discussion

The present results are the first to demonstrate a dynamic, circadian interaction between LA metabolism and the Warburg effect (e.g., glucose uptake and lactate production) that is both aligned and in balance with circadian-driven host factors under light/dark entrained conditions that contribute to the prevention of runaway tumor growth. Our findings lend support to the concept that tumor metabolic networks and fluxes are organized within a SCN-driven circadian time structure provided by the pineal nocturnal melatonin signal in the host. Disruption of the circadian organization of this host/cancer balance by LAN-induced melatonin suppression leads to a 24-hour a day constitutive hyperinsulinemia/hyperglycemia accompanied by elevated blood IGF-1 levels in the host and runaway constitutive metabolism and growth in the tumor. Inasmuch as melatonin suppresses insulin secretion while pinealectomy (e.g., melatonin removal) increases insulin resistance in rats, it is possible that the hyperglycemic response in circadian-disrupted animals was due to insulin resistance provoked by LAN-induced hyperinsulinemia secondary to nocturnal melatonin suppression.

The circadian rhythms in the Warburg effect and LA metabolism demonstrate that, rather than being static, these processes are dynamic over the 24-hour day. Their general alignment with one another also implies an important temporal relationship and biochemical interaction between LA metabolism to 13-HODE and aerobic glycolysis. The high rates of tumor LA uptake and metabolism to 13-HODE and aerobic glycolysis during the light phase drive a corresponding increase in tumor cell proliferative and survival activities whereas during the dark phase, these processes are repressed to become relatively, but not completely, quiescent as manifested by much lower cell proliferation and increased cell loss as manifested by diminished [^3^H]thymidine incorporation into DNA and DNA content, respectively. Thus, the net tumor growth rate in rats exposed to LD 12∶12 lighting conditions appears to reflect an overall balance in tumor circadian dynamics characterized by a crescendo in daytime metabolism, proliferation and cell survival alternating with a marked nighttime decrescendo in these processes. These findings also demonstrate that in the melatonin-suppressed state imposed by LAN, aerobic glycolysis and cAMP-regulated LA uptake and metabolism to 13-HODE operated at a persistently high level throughout the 24-hour day, ostensibly with support from LAN-induced host hyperinsulinemia, hyperglycemia in addition to high IGF-1 blood levels [28]. LAN-induced melatonin suppression disrupted this circadian-organized “yin and yang” of metabolic signal processing and proliferative activity leading to a 24-hour per day hyper-metabolic and -proliferative state that ultimately culminated in a robust net acceleration in the growth rate of tumor biomass. The fact that key signal transduction and transcriptional factors regulating tumor LA metabolism and the Warburg effect exhibit circadian oscillations *in vivo* that are completely disrupted by LAN suggests that these activities may play a unique role in regulating metabolic fluxes in coordination with classical allosteric feedback mechanisms controlling intermediary metabolism in human breast cancer xenografts.

There is a possibility that exposure to dim LAN rather than disrupting circadian rhythms of tumor metabolic, proliferative and signaling activities, via suppression of the melatonin amplitude, actually induced them to free-run independently of host rhythmicity, or led to the persistence of these rhythms in individual tumor cells that were unsynchronized with each other. These scenarios, however, are unlikely since the general circadian rhythmicity of the host was still entrained by LD, 12∶12 under dim LAN conditions whereas nocturnal melatonin was suppressed (Fig. 1A–C). Furthermore, the tumor perfusion experiments clearly demonstrated that melatonin directly suppressed the metabolic and proliferative activities of these xenografts (see discussion below). Moreover, our previous study in healthy human female volunteers demonstrated that the nocturnal circadian melatonin signal is directly responsible for suppressing proliferative and metabolic activities in human breast cancer xenografts acutely perfused with blood collected during the mid-dark phase [12]. A brief exposure of these volunteers to LAN induced melatonin suppression sufficient to negate the tumor suppressive effects of nocturnally-collected blood perfused through the xenografts [12]. It is also possible that the clock genes *Clock*, *Cry1* and *Bmal1*, which are involved in the circadian control of whole-body glucose metabolism [31], may have contributed to the circadian regulation of tumor glucose metabolism in our investigation. We recently reported that in spite of their failure to express the tumor suppressor and clock gene *Per2*, our tissue-isolated human breast cancer xenografts exhibit daily oscillations in the expression of *Clock, Cry1 and Bmal1* that were disrupted in the absence of a circadian melatonin signal [32]. Taken together with our previous results, the present findings make a strong argument for the nocturnal circadian melatonin signal being the internal zeitgeber responsible for the circadian tumor rhythms in metabolism, signaling, proliferation, and clock gene expression that are abrogated by dim LAN-induced melatonin suppression.

**Figure 5:**
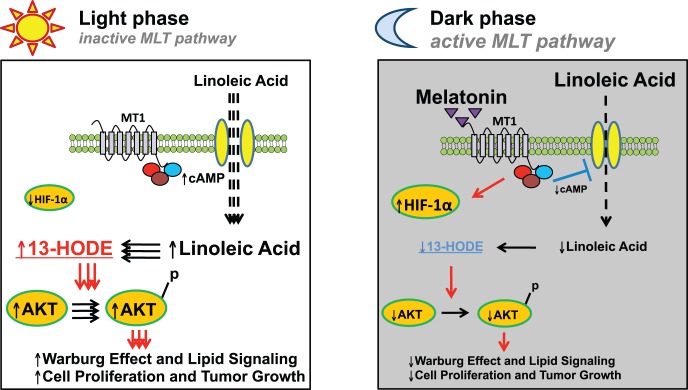
A provisional mechanistic scheme by which the host circadian system regulates cancer metabolism by linking lipid signaling with the Warburg effect in human breast cancer xenografts via the nocturnal melatonin signal. During the light phase, host blood levels of linoleic acid are low and melatonin concentrations are nil; however, cAMP-stimulated tumor uptake of linoleic acid and its metabolism to the mitogenic signaling molecule 13-HODE is maximal. Increased production of 13-HODE stimulates increased levels and phosphorylation/activation of AKT leading to enhanced aerobic glycolysis, cell proliferation and tumor growth. The introduction of dim LAN induces the continued operation of the tumor growth stimulatory mechanisms observed during the light phase. During the dark phase, host blood levels of linoleic acid are high and melatonin concentrations are maximal. Melatonin, by acting through tumor MT_1_ receptors [12], [27], down-regulates cAMP formation and blocks linoleic acid uptake and its metabolism to 13-HODE. Decreased production of 13-HODE results in an attenuation of AKT signaling leading to diminished aerobic glycolysis, cell proliferation and tumor growth. Although the exact role of melatonin-induced HIF-1α expression during the dark phase is unclear, it may involve newly discovered transcription-independent mechanisms that paradoxically contribute to the inhibition of cell proliferation and cell survival [32]–[34].

As indicated above, melatonin blocks the tumor production of 13-HODE by obstructing cAMP-dependent LA uptake via melatonin receptors [10], [12]. The present results extend those findings and suggest that melatonin potentially suppressed the Warburg effect, in part, by reducing 13-HODE formation and thus 13-HODE’s ability to activate AKT; however, more direct but currently unknown melatonin-mediated mechanisms may have also been involved. These results are supportive of our hypothesis that during the circadian light phase, abundant levels of tumor 13-HODE activate AKT to help drive the Warburg effect while during the circadian dark phase, melatonin decreases AKT phosphorylation/activation of this metabolic process by suppressing 13-HODE formation (Fig. 5). Interestingly, even the short-term tumor perfusion with either light phase-collected control blood (e.g., low melatonin) or blood containing a physiological nocturnal concentration of added melatonin produced results that appeared to mimic those obtained following long-term LD exposure in the circadian study. The failure of dim LAN exposure to negate the circadian expression of c-MYC observed under LD,12∶12 conditions coupled with melatonin’s inability to affect c-MYC expression during tumor perfusion suggests that melatonin is not involved in the circadian regulation of this transcription factor under these experimental conditions.

Considering the fact HIF-1α promotes the Warburg effect, it is somewhat problematic to mechanistically reconcile melatonin-induced stimulation of HIF-1α expression during either the circadian dark phase or tumor perfusion when aerobic glycolysis was actually diminished. Since HIF-1α expression is induced under hypoxic conditions [1]–[6] and melatonin significantly reduced tumor O_2_ uptake, it may be that melatonin stimulated HIF-1α expression by inducing tumor hypoxia during the dark phase under circadian-regulated conditions. Considering the fact that HIF-1α inhibits mitochondrial pyruvate dehydrogenase (PDH) [1], it is possible that a melatonin-induced increase in HIF-1α may have inhibited PDH during the glycolytically quiescent dark phase. This may serve to limit the entry of pyruvate-derived glycolytic carbons into the mitochondrial tricarboxylic acid cycle for the biosynthesis of cellular intermediates. Such a molecular timing mechanism might allow pyruvate levels to accumulate during the dark phase so that sufficient stores of this carbon source were readily available for lactate formation during the subsequent highly glycolytically active light phase of the next 24-hour period. It is also possible that elevated HIF-1α expression during the night, and in response to melatonin perfusion, may have paradoxically contributed to the inhibition of cell proliferation and cell survival [33]. This could have occurred via newly discovered transcription-independent mechanisms of HIF-1α action involving both its direct suppression of DNA replication [34] and/or its ability to functionally counteract the action of c-MYC by displacing c-MYC binding from the promoter of cell cycle kinase inhibitor p21*^cip1^* thus leading to its de-repression culminating in cell cycle arrest [35].

In view of the fact that HIF-1α expression can also be induced independently of hypoxia through the increased growth factor activation of the PI3K/AKT pathway [1]–[6], it is conceivable that under circadian-disrupted, low-melatonin conditions, HIF-1α expression may have been secondarily up-regulated by a hypoxia-independent LEN-induced increase in the activity of the PI3K/AKT in response to IGF-1. Such a potential mechanism is suggested by our recent observation that LAN increased circulating IGF-1 levels in host animals is accompanied by a corresponding increase in these xenografts of IGF-1 receptor (IGF-1R) expression and activation of the AKT-stimulatory kinase, phosphoinositide-dependent kinase 1 (PDK1) [28]. Thus, in response to LAN-induced melatonin suppression, the circadian disrupted and up-regulated pattern of HIF-1α over the 24-hour day in response to activation of the IGF-1/IGF-1R/PDK1/AKT pathway, may have served a different purpose by recruiting HIF-1α to work, via its transcriptional activity, coherently with AKT to cause the Warburg effect to go into “hyper-drive” 24-hours per day. Additional follow-up studies will be required to further flesh-out the complexities of this putative mechanistic scheme.

The circadian organization and disruption of tumor metabolism revealed by our experiments, and their consequences for tumor growth, may represent a next generation hallmark of cancer with respect to cancer prevention [36]. Comprehensive knowledge of the circadian nature of tumor metabolic signaling mechanisms and proliferative activity in the context of the circadian control of whole-body metabolism, may be essential for the rational development of new preventative and therapeutic approaches directed at critical metabolic targets [37], [38] that oscillate predictably throughout the 24-hour day. Therefore, the success of metabolically-targeted preventative and therapeutic strategies may ultimately depend upon their optimal temporal coordination with host/cancer circadian timekeeping. Moreover, LEN-induced circadian disruption of the host/cancer metabolic balance may contribute to an increased breast cancer risk in women working night shifts [20], [39] who may also be predisposed to developing a spectrum of metabolic diseases such as type-2 diabetes, obesity and/or metabolic syndrome [18], [19] that may further exacerbate breast cancer risk, as well as, compromise efforts to treat and/or further prevent breast and other cancers.
